# Supplementary material for: Trust and transparency in times of crisis: Results from an online survey during the first wave (April 2020) of the COVID-19 epidemic in the UK
Source: PLoS One. 2021 Feb 16;16(2):e0239247. doi: 10.1371/journal.pone.0239247 (PMC7886216; doi:10.1371/journal.pone.0239247)
Supplement: S1 File — (PDF) [file pone.0239247.s001.pdf]

LSHTM_Coronavirus_and_Health_Survey

Thank you for agreeing to take part in this survey, which is being carried out by researchers from the **London School of Hygiene and Tropical Medicine (LSHTM)**.

We are asking people to take part in our research in order to help us to understand how well different people around the UK are coping with some of the stressful events that are taking place during the current outbreak of coronavirus (COVID-19).

In this survey we will ask you to answer a series of questions about what you are doing and how you feel about what is happening during the outbreak. We are also interested in trying to understand how information about the disease is shared in communities

About This Study

- This research has received approval from the **LSHTM research ethics committee** (Reference 21846)
- The scientists leading this work are **Dr Shelley Lees** and **Dr Chrissy Roberts**.
- This study is **anonymous**, which means that we will neither know who you are, nor keep any information about your precise location.
- You do not have to answer all the questions if you do not wish to.
- The survey will take around 15-20 minutes to complete.
- The red line at the top of the screen shows your progress through the survey.

We are hoping to get as many people as possible across the UK to fill in this survey, so please send the link https://enketo.lshtm.ac.uk/::wsqM5QPj to your friends, families and co-workers.

We will share summary data from our study as we go along and this will be public data. You can view the RESULTS HERE

More information and detailed analysis are available on our project website.

*
To take part in this study, please confirm that you

- live in the United Kingdom
- are aged 13 years or older
- have read the information on the previous page
- understand that all data will be anonymous
- understand that the data from this survey will be shared publicly

Please click "OK" to give your consent to take part in this study

○ OK

Q1. What is your gender?

- ◯ Female
- ◯ Male
- ◯ Transgender
- ◯ Gender fluid or non-binary
- ◯ Another gender
- ◯ Prefer not to say

Q2. What is your ethnic group?

- ◯ White
- ◯ Asian/Asian British
- ◯ Black / African / Caribbean / Black British
- ◯ Mixed (White and Black)
- ◯ Mixed (White and Asian)
- ◯ Mixed (Any other mixed/multiple ethnic background)
- ◯ Arabic
- ◯ Another Ethnic Group
- ◯ Prefer not to say

Q3. What is your age

- ◯ 13-19
- ◯ 20-24
- ◯ 25-29
- ◯ 30-34
- ◯ 35-39
- ◯ 40-44
- ◯ 45-49
- ◯ 50-54
- ◯ 55-59
- ◯ 60-64
- ◯ 65-69
- ◯ 70-74
- ◯ 75-80
- ◯ Over 80

Q4. How many people live in your home?

- 1
- 2
- 3
- 4
- 5
- 6
- 7
- 8
- 9
- 10 or more

Q5. Where do you live?

- House
- Flat
- Assisted Living
- Residential Care Home
- Prison
- Hospital / Hospice
- Hotel/Hostel/B&B
- Shared living (student halls/shared houses)
- Another type of home

Q6. Do you have a garden?

- Yes
- No

Q7. Do you share your home with school aged children?

- Yes
- No

Q8. Do you regularly look after children (including grandchildren)

- Yes
- No

Q9. Do you have carer responsbility for older adults?

- Yes
- No

Q10. Which of the following things are you doing at the moment?

	Yes	No
**Seeing fewer people (Social Distancing)**	◯	◯
**Self-Isolation / Staying at home**	◯	◯
**Quarantining yourself** *i.e. Because someone in household has signs of COVID-19*	◯	◯
**Stockpiling food**	◯	◯
**Trying to quit smoking**	◯	◯
**Doing regular exercise to keep fit**	◯	◯
**Keeping in touch with your neighbours and community**	◯	◯
**Community action to support people affected by COVID-19**	◯	◯
**Spending quality time with your loved ones**	◯	◯
**Using exercise equipment in your home**	◯	◯
**Online keep-fit classes/videos** *For example "Coach Joe"*	◯	◯
**Strength and Flexibility Exercises**	◯	◯
**Yoga / Pilates**	◯	◯
**Walking/Running/Cycling Outside**	◯	◯
**Visiting a public gym or swimming pool**	◯	◯

Q11. Do you think that you have had COVID-19?

- ◯ I don't think I have had COVID-19
- ◯ I think I had COVID-19 but have now recovered
- ◯ I think I currently have COVID-19

Did you have a test which confirmed that you had/have COVID-19

- ◯ Yes
- ◯ No
- ◯ I don't know

How severe were/are your symptoms?

- ◯ Mild (I felt a little unwell)
- ◯ Moderate (I felt quite unwell)
- ◯ Severe (I felt extremely unwell)
- ◯ Very severe (I was hospitalised)

How many days were you sick for?

- 1 day
- 2 days
- 3 days
- 4 days
- 5 days
- 6 days
- 7 days
- 8 days
- 9 days
- 10 or more days

Q12. Including you, how many people in your home have had COVID-19?

- 0
- 1
- 2
- 3
- 4
- 5
- 6
- 7
- 8
- 9
- 10 or more

Q13. How many households in your local community do you know in which at least one person has had a coronavirus infection?

Do not include your own household.

- 0
- 1-2
- 3-5
- 6-10
- 11-20
- 21-30
- 30 or more

Q14. Where do you get your information on the coronavirus pandemic?

Select all that you use

	Yes	No

**Television news (including streamed news)**		
**Newspapers/Magazines**		
**People you work with**		
**Friends/ Neighbours/Family**		
**Facebook (public/friend pages)**		
**Facebook (private groups)**		
**Faith leaders**		
**Headteachers / Community leaders**		
**The Internet / Google Searches**		
**Twitter**		
**Whatsapp groups**		
**Radio**		
**Newsletters / Community groups / Employer updates**		

Q15. How trustworthy do you think each of these news sources is?

Score each from 0 (not trustworthy) to 5 (very trustworthy)
If you are unsure about anything, please leave it blank

	0	1	2	3	4	5
**Television news (including streamed news)**						
**Newspapers/Magazines**						
**People you work with**						
**Friends/ Neighbours/Family**						
**Facebook (public/friend pages)**						
**Facebook (private groups)**						
**Faith leaders**						
**Headteachers / Community leaders**						
**The Internet / Google Searches**						
**Twitter**						
**Whatsapp groups**						
**Radio**						
**Newsletters / Community groups / Employer updates**						

Q16. Do you think that your government is making good decisions about how to control COVID-19?

- Yes
- No

Q17. Do you think that the government tells you the whole truth about coronavirus and COVID-19?

- Always
- Mostly
- Sometimes
- Almost never
- Never
- I don't know

Briefly describe what it is that you think the government is not being fully truthful about?

Q18. Have you heard anything about coronavirus or COVID-19 that you think is being kept secret, covered up or hidden from the general public?

- Yes
- No

Briefly describe what you've heard

Feel free to add more than one thing

Where did you hear this information?

Select as many as you like

- ☐ Friends/ Neighbours
- ☐ Faith leaders
- ☐ Members of congregation
- ☐ The Internet
- ☐ Twitter
- ☐ Radio
- ☐ Facebook (private groups)
- ☐ At school / college / university
- ☐ Television news (including streamed news)
- ☐ Whatsapp groups
- ☐ Other
- ☐ Newspapers/Magazines
- ☐ Headteachers / Community leaders
- ☐ People you work with
- ☐ Family Members
- ☐ Facebook (public/friend pages)

Q19. Have you heard of people doing interesting, unusual or different things to fight coronavirus.

- ○ Yes
- ○ No

Please explain what they've been doing

Feel free to add more than one thing

Q20. Do you think that the government cares more about people or the economy?

- ○ Don't know
- ○ They care more about the economy
- ○ They care more about people and their health
- ○ About the same

Q21. If you were in charge, what would you do to control the outbreak?

Q22. Would you be willing to try an experimental vaccine if one became available?

- ◯ Yes
- ◯ No

Q23. Because of a health or memory problem do you have any difficulty doing any of the following tasks

Tick all that apply

- ☐ Dressing
- ☐ Walking across a room
- ☐ Bathing
- ☐ Eating, such as cutting up food
- ☐ Getting in and out of bed
- ☐ Using the toilet, including getting up or down

Does somebody help you with the tasks that you have difficulty with?

- ◯ Yes
- ◯ No

Who helps you with these tasks?

- ☐ Family members who live with you
- ☐ Family members who visit you
- ☐ Friend / Neighbour
- ☐ Home help or care
- ☐ Someone from a voluntary organisation (Age UK etc)
- ☐ Someone not listed above

If help or care from family or friends was not possible would you require the support from care services/ home help or personal assistants?

- ◯ Yes
- ◯ No

Q24. Are you worried about feeling lonely or isolated during the COVID-19 outbreak?

- ◯ Not worried at all
- ◯ A little worried
- ◯ Worried
- ◯ Very worried
- ◯ Terrified

Q25. How are you staying in touch with friends and family?

- ☐ I have no contact with friends and family
- ☐ Visiting (face to face contact)
- ☐ Speaking on the phone
- ☐ Social Media (Facebook/Twitter/Instagram etc)
- ☐ Video-phone (skype, zoom, facetime etc)
- ☐ Postal letters
- ☐ Email

Q26. How many times have you spoken to friends and family (who do not live in your home) in the last week?

For instance when you had a spoken conversation by telephone, skype or video phone with someone who does not live with you

- ◯ 0
- ◯ 1-2
- ◯ 3-5
- ◯ 6-10
- ◯ 11-20
- ◯ 20-29
- ◯ 30-39
- ◯ 40-49
- ◯ More than 50

Q27. Please estimate how many text/imessage/whatsapp messages have you sent and received in the last 24 hours that had something to do with COVID-19?

Include messages you have sent and received in group chats

- ◯ None
- ◯ 1-10
- ◯ 21-50
- ◯ 51-100
- ◯ 101-200
- ◯ 201-300
- ◯ more than 300

Q28. In the past two weeks, how often have you felt down, depressed, or hopeless?

- ◯ Not at all
- ◯ Several days
- ◯ More than half the days
- ◯ Every day

Q29. How many days could you stay in the house without going out to get food?

- ◯ 1-3 days
- ◯ 4-7 days
- ◯ 7-14 days
- ◯ 14-21 days
- ◯ 21-30 days
- ◯ More than 30 days

Q30. If you rely on prescription medicine, how long will your current supply last?

- ◯ 1-3 days
- ◯ 4-7 days
- ◯ 7-14 days
- ◯ 14-21 days
- ◯ 21-30 days
- ◯ More than 30 days
- ◯ I do not rely on prescription medicines

Q31. Roughly what percentage of people in your community do you think will catch coronavirus at some point?

- ◯ 10%
- ◯ 20%
- ◯ 30%
- ◯ 40%
- ◯ 50%
- ◯ 60%
- ◯ 70%
- ◯ 80%
- ◯ 90%
- ◯ 100%
- ◯ I don't know

Q32. Do you think that it is acceptable for governments to force some people to change their behaviours in order to control COVID-19?

- ◯ Yes
- ◯ No

Q33. If you are going to catch coronavirus, do you think it would be better to get it now, or later?

- ◯ Now
- ◯ Later
- ◯ I don't know

Q34. How worried are you about the coronavirus outbreak?

- ◯ Not worried at all
- ◯ A little worried
- ◯ Worried
- ◯ Very worried
- ◯ Terrified

**Q35. On 23rd March 2020, the Prime Minister Boris Johnson announced a complete lockdown in the UK.
Tell us what you have been doing to help you cope during this difficult time?**

____

Q36. Before the outbreak began, what type of exercise did you regularly do?

- ◯ None
- ◯ Mild (e.g. walking short distances, doing DIY etc.)
- ◯ Moderate (e.g. A gentle workout, Digging the garden, Dancing)
- ◯ Vigorous (e.g. Running/Jogging/Hiking, Cycling, Weightlifting)

What type of exercise are you doing now?

- ◯ None
- ◯ Mild (e.g. walking short distances, doing DIY etc.)
- ◯ Moderate (e.g. A gentle workout, Digging the garden, Dancing)
- ◯ Vigorous (e.g. Running/Jogging/Hiking, Cycling, Weightlifting)

Q37. Do you smoke?

- ◯ No
- ◯ Yes
- ◯ I quit smoking

Q38. How would you rate your overall health?

- ◯ Very Good
- ◯ Good
- ◯ Fair
- ◯ Bad
- ◯ Very Bad

Q39. Do you have a medical condition that you feel would increase your risk of getting seriously ill if you were to catch coronavirus?

- ◯ Yes
- ◯ No

Please describe the medical condition that you feel puts you at increased risk.

____

Q40. Does anyone else in your household have a medical condition that you feel would increase their risk of getting seriously ill if they were to catch coronavirus?

- ◯ Yes
- ◯ No

Q41. Has a doctor ever diagnosed you with any of the following?

	Yes	No
**Type 1 Diabetes**	◯	◯
**Type 2 Diabetes**	◯	◯
**Lung disease**	◯	◯
**Cancer**	◯	◯
**Stroke**	◯	◯
**Heart disease**	◯	◯
**High Blood pressure (hypertension)**	◯	◯
**Obesity**	◯	◯

Q42. Before the outbreak began, did you regularly attend a place of worship?

- Buddhist Temple
- Church
- Hindu Temple
- Mosque
- Sikh Gurdwara
- Synagogue or Jewish Temple
- Another place of worship
- None

Q43. Before the outbreak began, did you regularly take part in a group activity (classes, volunteering, book groups etc)

- Yes
- No

Q44. What is your employment status?

- Full Time Job
- Part Time Job
- Student
- Homemaker / Stay at home
- Retired
- Self Employed
- Unemployed
- Unable to Work
- Jobseeker

Q45. Which of these best describes the work you do?

- Admin/Secretarial
- Education
- Science
- Healthcare
- Retail
- Hospitality
- Finance & Business
- Transport & Communications
- Manufacturing
- Agriculture & Energy
- Other Service
- Entertainment & The Arts
- Public Services & Infrastructure
- Government (national/local)
- None of these

Q46. What is your yearly household income?

Include pensions, benefits and money from paid work

- Less than £15,000
- £15,000 - £24,999
- £25000 - £39,999
- £40,000 - £59,999
- £60,000 - £99,999
- More than £100,000
- Prefer not to say

Q47. What is the highest level of education you have completed?

- Completed Primary School
- GCSE / O-levels
- A levels / Highers / International Baccalaureate
- Some university / College of education / technical or vocational school
- University (first) degree
- Post-graduate degree
- Don't know
- Prefer not to say

Q48. Please select the first letters from your postcode from this list

i.e if your postcode is SW1A 1AA, enter SW, for G1 1NQ, enter G

- ◯ AB Aberdeen
- ◯ AL St Albans
- ◯ B Birmingham
- ◯ BA Bath
- ◯ BB Blackburn
- ◯ BD Bradford
- ◯ BF British Forces
- ◯ BH Bournemouth
- ◯ BL Bolton
- ◯ BN Brighton
- ◯ BR Bromley
- ◯ BS Bristol
- ◯ BT Northern Ireland
- ◯ CA Carlisle
- ◯ CB Cambridge
- ◯ CF Cardiff
- ◯ CH Chester
- ◯ CM Chelmsford
- ◯ CO Colchester
- ◯ CR Croydon
- ◯ CT Canterbury
- ◯ CV Coventry
- ◯ CW Crewe
- ◯ DA Dartford
- ◯ DD Dundee
- ◯ DE Derby
- ◯ DG Dumfries and Galloway
- ◯ DH Durham
- ◯ DL Darlington
- ◯ DN Doncaster
- ◯ DT Dorchester
- ◯ DY Dudley
- ◯ E East London
- ◯ EC Central London EC

- EH Edinburgh
- EN Enfield
- EX Exeter
- FK Falkirk and Stirling
- FY Blackpool
- G Glasgow
- GL Gloucester
- GU Guildford
- HA Harrow
- HD Huddersfield
- HG Harrogate
- HP Hemel Hempstead
- HR Hereford
- HS Outer Hebrides
- HU Hull
- HX Halifax
- IG Ilford
- IP Ipswich
- IV Inverness
- KA Kilmarnock
- KT Kingston upon Thames
- KW Kirkwall
- KY Kirkcaldy
- L Liverpool
- LA Lancaster
- LD Llandrindod Wells
- LE Leicester
- LL Llandudno
- LN Lincoln
- LS Leeds
- LU Luton
- M Manchester
- ME Rochester
- MK Milton Keynes
- ML Motherwell
- N North London

- NE Newcastle upon Tyne
- NG Nottingham
- NN Northampton
- NP Newport
- NR Norwich
- NW North West London
- OL Oldham
- OX Oxford
- PA Paisley
- PE Peterborough
- PH Perth
- PL Plymouth
- PO Portsmouth
- PR Preston
- RG Reading
- RH Redhill
- RM Romford
- S Sheffield
- SA Swansea
- SE South East London
- SG Stevenage
- SK Stockport
- SL Slough
- SM Sutton
- SN Swindon
- SO Southampton
- SP Salisbury
- SR Sunderland
- SS Southend-on-Sea
- ST Stoke-on-Trent
- SW South West London
- SY Shrewsbury
- TA Taunton
- TD Galashiels
- TF Telford
- TN Tonbridge

- TQ Torquay
- TR Truro
- TS Cleveland
- TW Twickenham
- UB Southall
- W West London
- WA Warrington
- WC Central London WC
- WD Watford
- WF Wakefield
- WN Wigan
- WR Worcester
- WS Walsall
- WV Wolverhampton
- YO York
- ZE Lerwick

Q49. How did you hear about this survey?

- Family Members
- People you work with
- Friends/ Neighbours
- Newspapers/Magazines
- Facebook (public/friend pages)
- Facebook (private groups)
- Faith leaders
- Headteachers / Community leaders
- The Internet
- Twitter
- Whatsapp groups
- Television news (including streamed news)
- Radio
- Members of congregation
- At school / college / university
- Other

Thank you very much for completing this survey.

Don't forget to check the results. You'll find a link on the front page after you submit

Please share the link to this survey as widely as possible
Now press the submit button!
