# Supplementary material for: Trust and transparency in times of crisis: Results from an online survey during the first wave (April 2020) of the COVID-19 epidemic in the UK
Source: PLoS One. 2021 Feb 16;16(2):e0239247. doi: 10.1371/journal.pone.0239247 (PMC7886216; doi:10.1371/journal.pone.0239247)
Supplement: S1 Table — (PDF) [file pone.0239247.s004.pdf]

S1 Table: Participant opinions on UK government prioritisation of COVID-19 response to economy or people & their health. Odds ratios compared to those who thought that the priority was a balance of both.

		Perceived Government Priority					
		The Economy		People & Their Health		Don't know	
**Variable**	**Group**	**OR**	**p**	**OR**	**p**	**OR**	**p**
Region	East Midlands	1.01 (0.77 - 1.25)	0.928	1.32 (1.06 - 1.58)	0.036	1.14 (0.78 - 1.50)	0.460
	East of England	0.95 (0.75 - 1.15)	0.654	1.15 (0.92 - 1.38)	0.223	1.08 (0.77 - 1.39)	0.634
	London (ref)	-	-	-	-	-	-
	North East	1.2 (0.97 - 1.43)	0.110	1.01 (0.73 - 1.29)	0.939	0.88 (0.49 - 1.27)	0.529
	North West	1.22 (1.02 - 1.42)	0.051	0.96 (0.71 - 1.21)	0.755	0.92 (0.59 - 1.25)	0.622
	Northern Ireland	1.69 (1.18 - 2.20)	0.043	0.77 (0.03 - 1.51)	0.496	1.26 (0.44 - 2.08)	0.578
	Scotland	2.18 (1.94 - 2.42)	<0.001	1.08 (0.76 - 1.40)	0.647	1.54 (1.16 - 1.92)	0.025
	South East	0.97 (0.79 - 1.15)	0.709	1.23 (1.03 - 1.43)	0.046	0.83 (0.54 - 1.12)	0.215
	South West	1.11 (0.91 - 1.31)	0.313	1.06 (0.83 - 1.29)	0.601	0.98 (0.66 - 1.30)	0.917
	Wales	1.2 (0.96 - 1.44)	0.142	0.77 (0.45 - 1.09)	0.117	0.81 (0.39 - 1.23)	0.339
	West Midlands	0.88 (0.66 - 1.10)	0.262	1.28 (1.04 - 1.52)	0.049	0.85 (0.50 - 1.20)	0.369
Age	20-34	1.65 (1.40 - 1.90)	<0.001	0.66 (0.36 - 0.96)	0.005	1.02 (0.63 - 1.41)	0.916
	35-54	1.84 (1.66 - 2.02)	<0.001	0.79 (0.60 - 0.98)	0.017	1.05 (0.77 - 1.33)	0.703
	55-69	1.52 (1.35 - 1.69)	<0.001	0.94 (0.76 - 1.12)	0.502	1 (0.74 - 1.26)	0.998
	70+ (ref)	-	-	-	-	-	-
Education	Completed Primary School	2.01 (1.40 - 2.62)	0.026	1.11 (0.41 - 1.81)	0.778	1.12 (0.02 - 2.22)	0.84
	GCSE/O-levels (ref)	-	-	-	-	-	-
	A level/Higher	1.53 (1.27 - 1.79)	0.002	1.15 (0.88 - 1.42)	0.327	1.19 (0.77 - 1.61)	0.429
	Further education	1.36 (1.16 - 1.56)	0.003	0.9 (0.69 - 1.11)	0.335	1.09 (0.77 - 1.41)	0.584
	University (first) degree	2.1 (1.90 - 2.30)	<0.001	0.87 (0.66 - 1.08)	0.178	1.48 (1.17 - 1.79)	0.014
	Post-graduate degree	2.22 (2.02 - 2.42)	<0.001	0.76 (0.55 - 0.97)	0.012	1.34 (1.02 - 1.66)	0.073
Gender	Female (ref)	-	-	-	-	-	-
	Male	1.16 (1.04 - 1.28)	0.019	1.05 (0.91 - 1.19)	0.492	1.05 (0.85 - 1.25)	0.655
	All other genders	2.15 (1.48 - 2.82)	0.026	0.88 (-0.16 - 1.92)	0.813	1.88 (0.84 - 2.92)	0.233
Income	Less than £15,000	2.55 (2.30 - 2.80)	<0.001	0.72 (0.43 - 1.01)	0.026	1.64 (1.20 - 2.08)	0.026
	£15,000 - £24,999	2.05 (1.81 - 2.29)	<0.001	0.84 (0.58 - 1.10)	0.185	1.72 (1.30 - 2.14)	0.011
	£25000 - £39,999	1.82 (1.59 - 2.05)	<0.001	0.76 (0.51 - 1.01)	0.035	1.66 (1.27 - 2.05)	0.011
	£40,000 - £59,999	1.52 (1.29 - 1.75)	<0.001	0.85 (0.60 - 1.10)	0.197	1.47 (1.08 - 1.86)	0.053
	£60,000 - £99,999	1.35 (1.11 - 1.59)	0.012	0.83 (0.58 - 1.08)	0.161	1.26 (0.86 - 1.66)	0.267
	> £100,000 (ref)	-	-	-	-	-	-
Model corrected for all covariates							
