# Supplementary material for: Trust and transparency in times of crisis: Results from an online survey during the first wave (April 2020) of the COVID-19 epidemic in the UK
Source: PLoS One. 2021 Feb 16;16(2):e0239247. doi: 10.1371/journal.pone.0239247 (PMC7886216; doi:10.1371/journal.pone.0239247)
Supplement: S2 Table — (PDF) [file pone.0239247.s005.pdf]

S2 Table: STM Topics, expected topic proportions and summaries of thematic content

	Topic	Topic Summaries	ETP
T1	Equipment	• Availability of PPE for NHS staff<br>• Testing capacity<br>• Health System preparedness	0.179
T2	Extent of Truth	• The public don't need to know the whole truth<br>• Government withholds information to avoid panic/ in the public interest<br>• Reasons for lack of transparency	0.214
T3	Rationale/ Politics	• Reasons for policy and changes<br>• Politicisation of the COVID-19 response	0.100
T4	Numbers	• Numbers of deaths/ people infected with COVID-19	0.095
T5	Long term	• Long-term effects of COVID-19 and lockdown on British society/ economy	0.124
T6	Settings	• COVID-19 in care homes<br>• Lack of recording of care home deaths	0.181
T7	Implementation	• Lack of information around timing and choice of policies and regulations to control COVID-19	0.105
